# Embracing Uncertainty: How literary writing helps us change our minds

**DOI:** 10.3389/fpsyg.2022.1019987

**Published:** 2022-10-11

**Authors:** Kim Devereux

**Affiliations:** The Department of English and Creative Writing, University of Exeter, Exeter, United Kingdom

**Keywords:** literary novel, uncertainty, contradiction, paradox, mindfulness, cognitive dissonance, blindness, insight

## Abstract

Creative writing and cognitive neuroscience can jointly illuminate how literary writing can change our minds by enhancing our tolerance for uncertainty. From my perspective as a novelist, I will show how literary writing hijacks the mechanisms of day-to-day perception and orchestrates cognition to facilitate vividness of (imaginary) experience and insight. Drawing on examples from my novel and other research, I will discuss the role of uncertainty and literary devices such as contradiction and paradox in stymieing habitual assumptions while inviting the reader’s sensory imagination and conscious awareness, thereby creating an open space for insight. I hypothesize that literary writing promotes a form of dual cognition that involves both sensory experience and detachment, is therapeutic, and may share some of the benefits of mindfulness.

## Introduction

*A map is not the territory*

—Alfred Korzybski, Science and Sanity

I am a novelist who is currently completing a Ph.D. in Creative Writing which comprises a novel and a dissertation informed by neuroscience. Since the late 20th century, neuroscience has advanced our understanding of cognitive processes. Writers and artists also have insight into the workings of cognition because they hijack the mechanisms of day-to-day perception to bring their works to life. It seems extraordinary, if I stop to think about it, that I spend my day typing abstract symbols, which result in experiences for my readers. And sometimes, the experiences that novels or poems provide can be so affecting that they rival, even trump, real life.

My Ph.D. and this paper arise from the question of how this is possible and how literary writing can provoke insight. I believe that creative writing and cognitive neuroscience can jointly illuminate the power of literary writing to change readers’ minds. I am offering my thoughts here as a novelist, in the hope that they might inspire collaborations between writers and neuroscientists.

Iain McGilchrist, in *The Master and his Emissary* (McGilchrist, 2012) suggests that we have two fundamentally different cognitive capacities or ways of attending. One, associated with the left hemisphere, is good at handling concepts, planning, and focusing on parts or details. The other, predominantly associated with the right hemisphere, is characterized by a more open attention and a capacity for understanding complex processes and interconnectivity. Both ways of attending are essential to our social and professional lives.

McGilchrist argues that left hemispheric cognition has, however, come to dominate how we view and navigate the world. If it were the other way round, with the open attention taking the lead, and conceptual, parsing, and planning cognition becoming its servant, we would experience the world differently and we would act differently, creating a better and more balanced world.

From this point of view, my novel and neuroscientific dissertation could be regarded as drawing on different cognitive capacities in investigating how literature moves us. The novel by inquiring experientially and through the act of writing, the dissertation by drawing on neuroscience. Together they build a model of the novel-reader interaction as an intimate dialog.

The novel I am currently writing, **The Generosity of Darkness*,* was inspired by neurologist Oliver Sacks’ description of Professor John Hull’s uncurable blindness as a ‘dark, paradoxical gift.’ (Sacks, 2003) ‘Hull’, Sacks writes, ‘comes to feel a sense of intimacy with nature, an intensity of being-in-the world, beyond anything he knew when he was sighted. This is not just “compensation” he [Hull] emphasizes, but a whole new order, a new mode of human being. With this, he extricates himself from visual nostalgia, from the strain, or falsity, of trying to pass as “normal, “and finds a new focus, a new freedom. His teaching at the university expands and becomes more fluent, his writing becomes stronger and deeper; he becomes intellectually and spiritually bolder, and more confident. He feels he is on solid ground at last.’

What happened to Hull throws into relief the cognitive habits which determine how we attend to the world. Sight includes our ability to perceive discrete objects, allowing us to track them and to plan our actions in the world. This way of attending to the world has a somewhat separating effect. Sound is much more immersive; there is no obvious boundary that divides us from the music in a concert hall, birdsong in a forest, or the swallowing roar of a train at close quarters. Blindness, according to Hull, leads to him apprehending the world differently.

I wanted to write about this different way of being in the world, alongside studying the neuroscience of different cognitive capacities and the brain’s plasticity. I came to realize that books, in a sense, take the reader’s sight away; they determine not only *what* the reader perceives but *how*—with which cognitive capacity—it is processed. Literary writing has ways to keep us in the dark; blocking conceptual rat runs, allowing us to perceive the familiar in novel ways. By literary writing, I mean novels and poetry which feature devices such as novel metaphors, ambiguity, contradiction, and detailed sensory description. They invite the reader’s creative and active participation in meaning-making and promote a more open, less knowledge-based cognition.

However, the left brain’s narrower and more goal-orientated focus does not cease. It goes on, at times haltingly, within a context of the broader attention of the right brain. The latter is more rooted in bodily experience, more willing to entertain contradictions, and more curious about what is just beyond the spotlight of awareness.

I will show how reading literary writing orchestrates our cognition through excerpts from my novel and a discussion of uncertainty, contradiction, and a dual cognition that involves both sensory experience and detachment.

## The dual attention – dual cognition paradox

The following is an excerpt from the *The Generosity of Darkness*.

Agnes didn’t hesitate to read the lines, she read them fast, taking in the gist of them like an efficient clerk. It was an apology. He’d been thinking about her. He wanted to see her.

And then descriptions of how he used to feel about her – how he still felt about her – she skirted over these expressions of feeling, before they could do any harm.

When she’d finished, she regarded the lines:

The way the ink had flowed from pen to paper, word after word, line after line, like water, once released continues along its course. Then her eyes returned to, **I love you*.* There was nothing that distinguished those three words from the other words that he had written, words like *Nürnberg*, *fade*, *b*reath** or **hope*. *I love you** was just words, made of letters and yet, unlike the other words, *I love you* hurt.

A sensation of something running from her nostril. She touched the liquid with her fingers and looked. It was red. She regarded the blood, not understanding. She was feeling completely fine.

Agnes, the main protagonist, has received a letter from Peter, a man she loved deeply but who had left her 15 years earlier. She is trying—and failing—to detach from the meaning of the words, from how they make her feel.

Reading literary writing invites a paradoxical state; to slip into a character’s skin, feeling what they feel and yet also to remain detached and aware of ourselves. In this way, novels can play host to existential problems without the reader, unlike Agnes, becoming overwhelmed by them. It is like having a lucid dream, conscious of everything but with an underlying feeling of safety.

Novels offer a personal odyssey without the consequences that replying to such a letter in real might entail. Paradoxically, it is because novels affording us this detachment that they might change our lives, by altering the way we process the painful things that happen to us.

The process of writing, as opposed to reading, also involves a dual cognition; feeling a character’s sensations while maintaining the detachment that is necessary to find the words to describe the kind of loneliness that leaves an empty ache in the stomach, even after a hearty meal. Without the element of perspective, the content of experience is all there is, but with it, we are able to perceive in fine-grained detail, without getting lost.

I am suggesting that reading offers similar benefits to mindfulness, by cultivating an open monitoring attention. Mindfulness is thought to reduce stress, by enabling the practitioner to stand back from a situation while being in it at the same time (Farb et al., 2007). Longitudinal studies of mindfulness practice have revealed changes in brain activation patterns (Fox et al., 2014) to this effect.

I will now discuss in more detail how literary writing and other arts create perspective by drawing attention to their artifice. It is as if they sabotage the beholder’s suspension of disbelief while at the same time maintaining it. Here are some examples:

Magritte painted a work known as “The treachery of Images.” It depicts a pipe along with the words in French, ‘This is not a pipe.’

Shakespeare frequently reminds the audience that they are watching a play. In Henry V the chorus refers to the Globe theatre as ‘this wooden O’ (Prologue, 14). Hamlet’s play-within-a play also invites (self-)consciousness of watching a play. Jane Eyre in Charlotte Brontë’s eponymous novel addresses the reader directly; “Reader I married him.” (Brontë, 2007).

You can judge for yourself, dear reader, the effect of being reminded that you are reading, even though this text is not an enthralling novel.

In addition, novels can draw on devices such as framing narratives, jeopardy, close or omniscient point of view, and detailed description to modulate the ratio between being merged with experience and separate from it.

Paradoxically the open monitoring attention invited by detachment does not lessen the reader’s investment; on the contrary, it heightens their capacity for vivid experiencing and pleasure.

A study by Mukhopadhyay proposes a similar notion in relation to the esthetic experience of paintings with his dual phase oscillation hypothesis (Mukhopadhyay, 2014). It describes how paintings co-ordinate two different aspects of our cognition. The first, the absorption phase is dominated by the task-induced demands of information processing (an overall decrease in activity of DMN from baseline level), and the second phase consists of ‘introspective detached contemplation’ (increased activation of dMPFC and vMPFC). Mukhopadhyay argues that esthetic delight arises when a dynamic balance is struck between the two, and the beholder is simultaneously aware of looking at a work of art and sensorially experiencing it at the same time.

I am proposing that the benefits of dual attention (open monitoring attention plus vivid experiencing) lie in how content is processed. Conscious access theory, which was first postulated by Bernard J. Baars in 1988 as Global Workspace Theory, hypothesizes consciousness as a ‘gateway to brain integration, enabling access between otherwise separate neuronal functions.’ (Baars, 2002). In other words, the added dimension of awareness plays a pivotal part in making content available more widely across different brain systems. The theory suggests that experiences are processed differently than if they remained unconscious. I am suggesting that the element of reading *about* a difficult experience, rather than living it, introduces a sense of perspective and remove. This remove allows the reader to notice their own responses more closely and in greater detail than if they were experiencing something harrowing in actuality and in real time.

For example, the description of the death of a pet allows the reader to attend to the transition of the animal being aware, breathing, and warm to becoming a limp body. The description may take up an entire paragraph while the process in life may last only seconds—too fast to integrate. Reading about such an experience may also afford processing of past losses.

Literary novels, in this way, train us to attend in a form of dual attention which includes an open monitoring attention that notices the simulated content of the novel as well as the reader’s responses to it. This is one way in which novels orchestrate our cognition, allowing us to attend to life in high definition.

## Apprehending the world differently

There are plenty of phrases that hint at our efforts to reframe, conceptualize, and integrate the unexpected, or maybe they are just desperate attempts to make the bad stuff feel less bad, less out of our control.

Every cloud has a silver lining.
Look at the bigger picture.
At least you do not have cancer.
It is what it is.
It just wasn’t meant to be.
Everything happens for a reason.
Give it time (delivered with empathetic look or sigh).
And my all-time favorite: Shit happens.

After his wife died of cancer, author and lay theologian C.S. Lewis wrote a pamphlet called *A Grief Observed*. After many pages of trying to make sense of his wife’s death, he concludes, ‘Are not all these notes the senseless writings of a man who will not accept the fact that there is nothing we can do with suffering except to suffer it?’ (Lewis, 2015). The line has always stayed with me, as if it contained something far more useful than all the phrases quoted above.

The most striking aspect of John Hull’s description of his experience of blindness in his book *Touching the Rock* (Hull, 1990) is that he seems to be curious about what it is like to experience the world in this new and different way. He seems to find a new freedom not in spite of blindness but because of it. A surprising suggestion given how debilitating it is to live without sight.

As a writer, I aim to describe experiences in close sensory terms. For this reason, I blindfolded myself for 24 h or more at a time. This approach has limits as it is very different from losing your sight permanently. I spoke to blind people to gain insights into their experience.

I tended to go into meetings blind, in a literal sense, because I wanted to know what it is like to meet someone without knowing what they look like. The timbre, speed, and softness of their voice and the experiences they shared were all I knew. They told me of the physical dangers, the isolation, and how tiring it is to employ elaborate strategies to accomplish tasks which sighted people perform with ease and automation.

I felt guilty for driving myself to these appointments with the ease that sight affords and then relaxing into the generosity of not seeing and of not being seen. The process has changed me. I used to believe that the moment I saw a person I knew something about them, now I distrust my first impressions. In the past upon meeting, I would try get to know someone by asking questions but now I relish the feeling of not knowing a person when I first meet them.

All this contributed to my wanting to write about the space beyond the known and to ask if the familiar could become unfamiliar. The main protagonist of my novel, Agnes, is a ceramic artist in her 40s. She lives alone and has barely any friends. After losing her sight, a support worker teaches her how to navigate to the nearest shop.

Agnes was trailing her hand along the rough pebble-dashed surface of a wall. Sean was watching her. She’d made a decision not to tell him about the difficult weekend. She was practising the route to the corner shop. He’d promised not to intervene unless she was in danger. The wall was dirty and sharp in her hand. Was this the first or the second lot of pebble dash? Her hand dropped into empty space. The wall had ended. Was there another house to come or was this the last one before the crossing? Where was Sean? Probably just a few feet behind. She listened to the sound of the cars. The road sounded close but the noise seemed to be coming from multiple directions. It had to be the crossing she was looking for.

Sean had told her to use the cane for the last bit. She took it out of her bag and extended the telescopic segments. She grasped the handle and placed her palm briefly on the wall again to make sure she was facing in the right direction. Then she let go of the wall and began walking towards the sound of traffic, sweeping the cane in semi-circular movements across the pavement. The scraping sound of the metal tip offended her ears. It also announced her presence to everyone. That was of course part of its function, to flag to the sighted that a blind person was trying to make her way in their world.

She waited for the sensation of the ball-like blisters on the ground, marking out the pelican crossing. Suddenly, the cane dropped into a void and the roar of a lorry engulfed her, followed by a draft of air and grit. She’d got far too close to the road. But surely, Sean would have intervened if it had been unsafe, maybe she wasn't as close as she thought.

She found the edge of the kerb with the cane. She was right by the road. But where were those bumps so she could find the control panel for the lights? She took a few breaths and tried to calm herself following Sean’s advice. Couldn’t he see she was struggling?

The passage blinds the reader, they are afforded no map or omniscient perspective. They therefore share Agnes’ discomfort and shock on ‘the edge’. It is very disturbing to leave behind the familiar—‘where? … probably … surely … maybe’—but it has its rewards. As a result of my own experiences without sight I no longer so firmly believe that my way of seeing the world is definitive.

The unknown or unexpected is also important in the process of writing. I find it helpful to know what the novel is about, in this case; blindness as a dark, paradoxical gift, and to have a plan for the plot.

And yet when I started writing the scene I quoted at the beginning of this piece, the letter from Peter surprised me as much as it surprised Agnes. Perhaps, it was a postcard from my subconscious. The letter brought the themes of betrayal and loss of trust into the ‘theatre of awareness’ that is the novel. But it was only later that I realized that the earlier loss of Peter foreshadowed the later loss of blindness and that Agnes’ loss of seeing made it necessary for her to face that Peter’s betrayal of her shaped the way she saw the world.

At the end of this process, Agnes embraces uncertainty again, the way she did when she was younger. I hope that novels can help us do the same.

## Embracing uncertainty and contradiction

Why is it difficult for most of us to include uncertainty and contradiction in our outlook? And why would it help? In the words of Donald Tusk, the former president of the European Council, ‘*While the truth may be more painful, it is always more useful*.’ (Boffey and Jennifer, 2019) So why would we want to resist noticing all relevant information.

Predictive processing, a leading model of brain function in neuroscience, offers a useful way of looking at the underlying mechanism of perception. It explains why it is not easy for us to see something with ‘new eyes’, as the expression goes. Predictive processing suggests that the brain is constantly generating and updating our model of the world. Two processes are occurring in the brain: One is a top-down process which simulates the experience that is expected and the other is a bottom-up process based on the sampling of the sounds, sights, smells, and sensations from the environment. If the two do not match a prediction error is generated. It is either resolved by updating the model or by behaving in a way that brings reality into line with the model (‘active inference’).

While our perception of the world, thanks to predictive processing, is fast and efficient it is always partial. Also, it is sometimes difficult to notice information that is at odds with our beliefs or expectations. There is a certain pressure for our model of how things are to be coherent. Predictive processing is not designed to include contradictions because its purpose is to dish up a definitive version of reality in as close to real time as possible.

Leon Festinger’s cognitive dissonance theory (Festinger, 1957) points to similar mechanisms in the brain from the point of view of psychology. Karl Friston, a leading neuroscientist, recently commented on the parallels between predictive perception theory and cognitive dissonance theory (Friston, 2018). Cognitive Dissonance theory suggests that we constantly strive for cognitive consistency, trying to eliminate any contradictory aspects from our experiences, even in retrospect. Studies show that individuals not only choose something because they like it, but that they like something because they chose it. So, after making a difficult choice between two equally preferred items, participants—post the event of choosing—state that they now prefer the chosen item over the unchosen one (Brehm, 1956). The preference adjustment resolves the ‘cognitive dissonance’ of having to choose between two equally preferred options.

As for Agnes, to restore cognitive consistency after Peter unexpectedly left her, she concludes that Peter never loved her in the first place. This affects the way she lives from that point onward. She no longer believes in love. What would Agnes’ life have been like if she had been able to entertain the contradiction that she had been absolutely loved by Peter and yet left? Is it possible to hold contradictory notions in mind simultaneously?

It might seem that such cognition is unhelpful as it defies logic. On the contrary, it is important to learn to tolerate ‘messy meaning’ and uncertainty not in order to believe falsehoods but because a deeper truth might be glimpsed in the process.

## Reading sensory detail – from the map into the territory

Agnes’ support worker hands her an object:

Sean touched her hand, placing an object in it. It was the size and shape of a tennis ball and it was covered in a liquid. Through the moist layer she felt a bumpy surface which had some give in it. The coating, even though it felt wet, didn’t seem to stick to her hands. ‘What is it?’ she asked.

‘What do you think it is?’

‘I have no idea,’ she said.

‘What does it feel like?’

‘It’s round and it is covered in some kind of fluid.’

‘Do you like the feel of it?’

‘Yes, it’s almost soft.’ She said and lifted it to smell it.

‘No,’ he said and held back her hand.

‘Why?’

‘It’s an orange covered in mould.’

She put it on the table. ‘Uggh.’

He laughed. ‘You might want to wash your hands.’

She got up and found the sink quite easily. The water running over her hands felt very similar to the mould.

She turned around, hoping she was facing him, ‘I don’t know what point you’re trying to make but you’ve just proved my point.’

‘Which is?’

‘Some things can only be done by sight.’

‘That may be true. But wasn’t it interesting?’

‘No,’ she protested, even though she agreed with him.

Primary, sensory experience is a way of finding some freedom from the predictive aspect of perception, which might exclude ‘unimportant’ detail from our awareness. Before the object is identified, Agnes notices vividly a ‘moist layer’, ‘bumpy surface’, and that the object is ‘rounded’. But she is conflicted (‘No…even though’) about the value of having been deprived of the information that she is holding an orange. If she had known it was an orange, she could have deduced that the soft, moist sensation may indicate mold.

On the other hand, the absence of knowing, directs her attention to her fingertips and heightens her experience.

I am suggesting that Literary writing employs novel metaphors, contradiction, and detailed sensory description to de-throne the knowledge-based aspect of cognition, so we can experience afresh what our senses (or the senses of our imagination) are tasting. These devices get us from the map into the territory.

Can we live life with the kind of attention we give to an unknown object when our eyes are closed? Can we find a little freedom from the concepts, beliefs, and perceptual habits that shape the way we apprehend the world?

Writing occupies a special place in that it is situated at the nexus of symbols and experience. On the one hand, words direct our attention to particular aspects of an object, on the other they can block off well-worn conceptual grooves, forcing us off piste where we rely on the exact texture of the ground to find our bearings.

I used to make films. When I first started to write I missed having recourse to locations, music, and actors to bring my ideas to life. Then, it dawned on me that with ‘mere’ words I could command armies many thousand strong, control the density of fog and the path of a single tear.

Words are anything but ‘mere’; they are a powerful code that represents reality. Part of their power is that they are more open to interpretation than actual sounds and images. It is the use of this code that makes it possible for novels and readers to become true partners in creation.

In another passage from my novel, I was aiming to capture what it is like to relate to an unknown world primarily through sensory information. Agnes participates in a walk and for the first time:

Everyone’s footsteps and voices re-bounded from the walls of the canyon, giving her clues about the scale of her surroundings. Then the loud screeching of a bird brought even more definition. It was the equivalent of lightning illuminating a landscape at night. Then the world faded once more into absence.

She listened to the drawing of their breaths and the shuffling of their clothes as they walked. The noises seemed embarrassingly private – as if she, and only she, was privy to a secret and intimate world. The way Zora’s breath caught slightly towards the end of the inhale high up in her throat and Callum’s boots met the ground with certainty and Syddall cleared his throat every now and then…

The bird called again. The sound got inside her. What did it even mean ‘inside’ and ‘outside’?

Literary reading invites both attending to the map and the territory, as here in Agnes’s walk. Agnes’ blindness affords me as a writer the opportunity to describe the world in an unusual way, without the old way of parsing things, no longer split by the dualism of inside and outside, perhaps without firm boundaries at all.

Paradigm shifts are possible when habitual ways of seeing cease to be the only way.

I have described different cognitive capacities which are trained in the process of literary reading. This dialog between reader and text orchestrates left brain and right brain processing in a way that shifts cognition away from certainty and toward a more open form of attending which is more grounded in sensory detail.

As a medium, literary writing can do this because it interfaces with the reader’s brain in unique ways, promoting a style of cognition which facilitates integration of difficult life experiences and promotes insight and the updating of beliefs.

## Data availability statement

The original contributions presented in the study are included in the article/Supplementary material; further inquiries can be directed to the corresponding author.

## Author contributions

The author confirms being the sole contributor of this work and has approved it for publication.

## Conflict of interest

The author declares that the research was conducted in the absence of any commercial or financial relationships that could be construed as a potential conflict of interest.

## Publisher’s note

All claims expressed in this article are solely those of the authors and do not necessarily represent those of their affiliated organizations, or those of the publisher, the editors and the reviewers. Any product that may be evaluated in this article, or claim that may be made by its manufacturer, is not guaranteed or endorsed by the publisher.
